# SARS-CoV-2 and Atherosclerosis: Should COVID-19 Be Recognized as a New Predisposing Cardiovascular Risk Factor?

**DOI:** 10.3390/jcdd8100130

**Published:** 2021-10-10

**Authors:** Mattia Vinciguerra, Silvia Romiti, Giuseppe Massimo Sangiorgi, David Rose, Fabio Miraldi, Ernesto Greco

**Affiliations:** 1 Department of Clinical, Internal Medicine, Anesthesiology and Cardiovascular Sciences, Sapienza University of Rome, 00161 Rome, Italy; fabio.miraldi@uniroma1.it (F.M.); ernesto.greco@uniroma1.it (E.G.); 2 Division of Cardiology, Department of Biomedicine and Prevention, Cardiac Cath Lab, University of Tor Vergata, 00133 Rome, Italy; gsangiorgi@gmail.com; 3 Lancashire Cardiac Centre, Blackpool Victoria Hospital, Blackpool FY3 8NP, UK; davidrose@libero.it

**Keywords:** COVID-19, SARS-CoV-2, atherosclerosis, cardiovascular disease, inflammation

## Abstract

At the beginning of the COVID-19 pandemic, the lung was recognized as the main target organ; now, new evidence suggests that SARS-CoV-2 infection leads to vascular disease. In a previous review, we supposed a bidirectional link between endothelial dysfunction and COVID-19, identifying atherosclerosis as having a crucial role in its pathogenesis. Atherosclerosis with an existing endothelial dysfunction may worsen COVID-19 manifestations, leading to adverse outcomes, as largely reported. However, COVID-19 may be the trigger factor in the progression of the atherosclerotic process up to making it clinically manifest. The thrombotic complications can involve not only the atherosclerotic plaque, but also the durability of the surgical device implanted to treat a pre-existing coronary artery disease as recently reported. The burden of the disease makes necessary a long-term stratification of patients, revising drastically targeted therapy among others.

The global pandemic has resulted in 231,614,451 confirmed infected patients, who were affected by severe acute respiratory syndrome coronavirus 2 (SARS-CoV-2), as of 26 September 2021 [1]. Coronavirus disease 2019 (COVID-19) includes a spectrum of clinical manifestations, ranging from completely asymptomatic to multiorgan failure. 

The high clinical variability is associated, among others, with the different expression of the binding receptor angiotensin-converting enzyme 2 (ACE2) on the surface of endothelial cells. Apoptosis and cellular damage are the main consequences of the vascular endothelial cell infection. In addition to the direct viral infection, the destabilization of ACE2, caused by the spike-binding glycoprotein (S protein), produces a deregulated activation of the renin–angiotensin system, leading to endothelial dysfunction [2].

Lei et al. [3] confirmed, through animal models, the ability of the spike glycoprotein of SARS-CoV-2 to cause endothelial injury, that leads to mitochondrial dysfunction and impairs the synthase of endothelial nitric oxide. 

At the beginning of the pandemic, our group published an article that suggested a bidirectional link between SARS-CoV-2 and atherosclerosis [4]. Atherosclerosis is triggered by the chronic inflammation of the endothelium, leading to a disorder in the delicate homeostasis of the endothelial cells’ properties, such as a hemostatic balance, inflammatory response, and vasomotor tone, amongst others. The underlying endothelial dysfunction might represent the ideal deregulated immunological setting in which SARS-CoV-2 triggers a ‘‘cytokine storm’’ with severe clinical manifestations. 

The subsequent inflammatory response to the exacerbation of a pre-existing endothelial dysfunction may explain the different inter-individual susceptibility to the viral infection, potentially leading to adverse outcomes; indeed, predisposing cardiovascular conditions, such as hypertension, diabetes mellitus type II, and coronary artery disease, are known to increase the patients’ vulnerability and, thus, the risk of mortality due to COVID-19 [5]. 

On the other hand, the hyper-inflammatory response leads to the thromboinflammatory one, potentially favoring and accelerating the atherosclerotic process leading towards adverse clinical manifestations. Atherosclerotic plaque rupture is strongly associated with excessive amounts of inflammatory cytokines. Indeed, an increased production of IL-6, TNF- α, and IL-1 α may result in protease activation, causing a transition from a stable to a pathological atherosclerotic injury and the degradation of the plaque protective fibrous cap. 

## 1. The COVID-19 Thromboinflammatory Response and the Immunopathological Connection with Atherogenesis

The pathogenicity of SARS-CoV-2 is unbreakably linked to the magnitude of the systemic release of pro-inflammatory cytokines [6]. A ‘‘cytokine storm’’ is caused by the maladaptive host immune response to SARS-CoV-2 with an excessive production of CD14++ and CD16+ inflammatory monocytes, exacerbated by the hyperactivation of the T helper (Th) 1 lymphocyte pathway [7]. The aberrant activation of the innate immune pathways causes a complex multiorgan disorder with thromboinflammatory manifestations; at a histological exam, infected tissues are infiltrated by large amounts of monocytes and neutrophils with thrombotic microangiopathy (TMA) as an expression of microvascular endothelium involvement [8]. The hypercoagulable state with increased liver production of acute-phase proteins (APPs) is mainly triggered by cytokines, interleukin 6 (IL-6), among others, of which circulating amounts correlate with disease severity [9,10].

The derailed inflammatory response, with growing and consistent evidence supporting the central role played by complement dysregulation, found as an early finding in the COVID-19 natural history, causes hyper-inflammation, immunothrombosis, and microvascular endothelium injury [11,12,13,14].

The delicate balance of complement system activation, regulated to perfectly counteract viral infection, may lead to the inadequate control of SARS-CoV-2 when compromised [15].

Different pathological studies have observed a complement deposition in various tissues of deceased COVID-19 patients with a peculiar involvement of lung cells, with an in vitro study that, interestingly, observed an elevated deposition in lung cells rather than in healthy controls, non-dependent of ACE2 overexpression [16]. Specifically, the membrane attack complex (MAC or C5b-C9) mediated injury, as seen in complement-mediated disease, was shown, identifying endothelial cell abnormalities typical of a TMA [11,17,18].

Consistent with an increasing severity of disease conferred by complement system over-activation, less lung inflammation and injury have been demonstrated in C3-deficient mice with SARS-CoV-2 infection, although showing a similar viral load as control wild-type mice [19].

Elevated serum C5a, a serine protease meaningful for the proinflammatory complement-mediated response, represented a potential biomarker in predicting the severity of disease [17,20].

The upregulation of this latter protease triggers thromboinflammation with the recruitment and priming of neutrophils in a process called neutrophil extracellular trap (NET) release or NETosis; a potent activator of cultured endothelial cells in COVID-19, potentially leading to microvasculature occlusion. As well as circulating complement components, NETosis serum levels positively correlate with disease severity due to the cytotoxic effect against epithelial and endothelial cells [15].

The dysregulated interplay between NET and complement, which leads to an aberrant immune responsiveness, has been recently evaluated in COVID-19 by Skendros et al. [21] finding that selective complement inhibitor drugs may decrease and attenuate neutrophils activation and NETosis [15].

Besides the involvement in severe COVID-19, NETs may play a decisive role in atherogenesis with a multitude of implications. The beneficial cholesterol efflux capacity conveyed by high-density lipoprotein (HDL) particles may be reduced due to oxidative stress induction. Platelet adhesion, activation, and aggregation may be stimulated by NETs, which further promote the accumulation of prothrombotic molecules such as von Willebrand factors and fibrinogen [22].

On the other hand, the complement system, the hallmark of severe COVID-19 manifestations, has showed, besides plasma levels, a high local deposition associated with arterial intimal thickenings. C5b-9 deposition on endothelial cells promotes the release of thrombotic factors, triggering inflammatory cytokines production [23].

In this setting, early atherosclerotic plaques reflect as an early immunopathological alteration in the activation of the complement system, potentially tracked by increasing circulating levels of C5, which correlates positively with the global plaque volume and coronary calcification [24].

## 2. Clinical Insights

The reported cases of acute myocardial infarction and the spontaneous dissection of coronary arteries highlight the exuberant inflammatory and proteolytic activity in weakening vessel walls in patients affected by severe manifestations of COVID-19 [25,26,27]. This might explain why patients who have undergone surgical or interventional management and contracted SARS-CoV-2 could be more prone to vessel luminal thrombosis. In our review [4], we postulated that percutaneous coronary intervention in the management of acute coronary syndrome might fail due to the high frequency of stenting thrombosis. In this context, a higher than expected number of stent thrombosis was reported in patients affected by COVID-19 after the implantation of drug-eluting stents [28,29]. Similarly, a coronary artery bypass graft might be complicated by early thrombosis, exacerbated by the excessive deregulation of the endothelium, particularly for a graft constructed using a vein conduit. The influence of COVID-19 on the patency rates of coronary artery bypass grafts may represent a catalytic factor in the progression of thrombosis. Other than the thrombotic complications, we believe that the magnitude of the inflammatory responses triggered by COVID-19 may adversely impact left ventricular remodeling after acute myocardial infarction. Recent evidence suggests that left ventricular remodeling depends on the pro-inflammatory state of the individual patient [30]. Furthermore, the prevalence and the prognostic value of pulmonary hypertension and right ventricular dysfunction in COVID-19 patients have been investigated by Pagnesi et al. [31] in a single-center observational study. Interestingly, only 4% of the sample population had the concomitance of the two conditions. It has been demonstrated that in the group of patients with a right ventricular dysfunction, there is a strong association with pre-existing cardiovascular comorbidities such as a myocardial infarction and coronary artery by-pass graft. Interestingly in the same group, there is no correlation with the severity of the infection. Therefore, the right ventricular dysfunction might represent the clinical evolution of an exacerbation of the pre-existing cardiac diseases.

Conversely, pulmonary hypertension is strongly associated with a severe disease progression, showing a higher incidence of all-cause mortality.

These findings show that an endothelial dysfunction may be responsible for an early and more severe presentation of several clinical conditions. The complexity underlying the biological pathways involved makes us believe that we are just observing the tip of the iceberg.

A complement system dysregulation and over-activation, followed by neutrophils priming and cytokines cascade, may promote the atherosclerotic progression starting from its subclinical phase. Therefore, we believe the long-term effects of COVID-19 on the cardiovascular system, with an increase in the incidence of coronary artery disease, could be predictable.

This future perspective and the increasing number of cases make it necessary to plan tailored preventive measures. The correct stratification of patient’s risks is crucial.

## 3. Future Perspectives

A great future challenge could arise from the significant difference in COVID-19 vaccine administration around the world. Indeed, as of 26 September 2021, only 2.2% of people in low-income countries have received at least one dose [32]. The numbers of COVID-19 vaccine doses administrated in African countries are the lowest, followed by South America, and Asian countries. COVID-19 is associated with worse outcomes in minority and marginalized communities due to the socio-economic status, life-style choices, and biological factors [33,34,35]. For this reasons, vulnerable groups, ideally, would benefit from preventive measures to avoid a new pandemic of cardiovascular disease. As atherosclerosis and COVID-19 share similar biological pathways, targeted therapy has been focused on counteracting the activation of key proteins. Although results may be encouraging, drugs that selectively target cytokines are still too expensive and far from being mass produced. In contrast, anti-platelet and anti-inflammatory drugs such aspirin and statin therapy may have a protective role against thrombotic complications following COVID-19. In particular, statin therapy has demonstrated, beyond cholesterol lowering, to have pleiotropic properties. Newsworthy statin therapy for its immunomodulatory properties has shown beneficial effects in patients with auto-immune inflammatory diseases such as rheumatoid disease and systemic lupus erythematosus and a potential add-on therapy in various infectious diseases. Long-term statin use has proven to be beneficial in the setting of bacterial pneumonia and influenza. Fedson et al. [36] reported an association between outpatient statin treatment and a reduction in disease severity and mortality during the 2009 H1N1 pandemic. Recent findings highlighted a potential role of statin therapy in COVID-19 patients: a reduction in in-hospital mortality in patients with diabetes mellitus and COVID-19 has been reported [37]. Additionally, in a retrospective cohort of 7171 patients with COVID-19, statin use was independently associated with lower disease severity and intensive care unit admission [38]. The use of simvastatin alone or in combination therapy with ezetimibe has been observed to downregulate systemic immune activation during acute RNA viral infection [39].

In the field of cardiovascular disease, statin therapy is often administered even in the absence of dyslipidemia, taking advantage of its anti-inflammatory effects in the subclinical phases of the atherosclerotic process. The reduction in the circulating levels of the inflammatory markers is the mechanism that helps to slow down the disease, preventing the formation of atherosclerotic plaque. A potential role of statin in plaque stabilization has also been reported [40].

In this context, statin therapy may be recognized as a valid treatment in the long-term management of COVID-19 patients, in order to prevent the atherosclerotic spread, especially in the elderly with cardiovascular risk factors independently on cholesterol serum levels. Subclinical atherosclerosis largely affects middle-aged individuals, often asymptomatic, and a tailored identification of more vulnerable patients who undergo COVID-19 is necessary. As seen afore, circulating biomarkers such as C5a plasma levels and the biomarker of NETosis activation may help in identifying an increased risk for atherogenesis and atherosclerotic plaque progression.

These marker assays may be helpful in the decision to prescribe statin therapy for middle-aged individuals affected by COVID-19, with a potential role in adding more statins in individuals who have already assumed therapy.

The risk stratification allows us to identify another category of patients who are particularly vulnerable to COVID-19, related to cardiovascular complications. Patients affected by SARS-CoV-2 infection, who underwent or are prone to go through the surgical management of coronary artery disease, would benefit from a tailored antiplatelet therapy. Indeed, in patients with a previous history of interventional procedures, COVID-19 may be a triggering factor of luminal thrombosis; thus, requiring different regimens of antiplatelet drugs.

In conclusion, we believe that COVID-19 could be recognized as a new risk factor for cardiovascular diseases. Revised measures of prevention and management should be required. Preclinical studies are, firstly, necessary in assessing the impact of COVID-19 on the cardiovascular system, with a comprehensive evaluation centered on atherosclerotic plaque progression and the long-term dosage of circulating inflammatory proteins. The effect of statin therapy in modulating the systemic immune response against viral infection, as seen in [39], needs to be supported by others pre-clinical evidence.
